# Experimental Models of Neuroimmunological Disorders: A Review

**DOI:** 10.3389/fneur.2020.00389

**Published:** 2020-05-12

**Authors:** Ana Paula Bornes da Silva, Rodrigo Braccini Madeira Silva, Leise Daniele Sckenal Goi, Rachel Dias Molina, Denise Cantarelli Machado, Douglas Kazutoshi Sato

**Affiliations:** ^1^Neuroinflammation and Neuroimmunology Laboratory, Brain Institute, Pontifical Catholic University of Rio Grande Do Sul (PUCRS), Porto Alegre, Brazil; ^2^School of Medicine, Graduate Program in Pediatrics and Child Health, Pontifical Catholic University of Rio Grande Do Sul (PUCRS), Porto Alegre, Brazil; ^3^Research Center in Toxicology and Pharmacology, School of Health and Life Sciences, Pontifical Catholic University of Rio Grande Do Sul (PUCRS), Porto Alegre, Brazil; ^4^School of Medicine, Graduate Program in Medicine and Health Sciences, Pontifical Catholic University of Rio Grande Do Sul (PUCRS), Porto Alegre, Brazil; ^5^Molecular and Cellular Biology Laboratory, Brain Institute, Pontifical Catholic University of Rio Grande Do Sul (PUCRS), Porto Alegre, Brazil

**Keywords:** immune system, autoantibodies, demyelination, neuroimmunological diseases, preclinical models

## Abstract

Immune-mediated inflammatory diseases of the central nervous system (CNS) are a group of neurological disorders in which inflammation and/or demyelination are induced by cellular and humoral immune responses specific to CNS antigens. They include diseases such as multiple sclerosis (MS), neuromyelitis optica spectrum disorders (NMOSD), acute disseminated encephalomyelitis (ADEM) and anti-NMDA receptor encephalitis (NMDAR encephalitis). Over the years, many *in vivo* and *in vitro* models were used to study clinical, pathological, physiological and immunological features of these neuroimmunological disorders. Nevertheless, there are important aspects of human diseases that are not fully reproduced in the experimental models due to their technical limitations. In this review, we describe the preclinical models of neuroimmune disorders, and how they contributed to the understanding of these disorders and explore potential treatments. We also describe the purpose and limitation of each one, as well as the recent advances in this field.

## Introduction

**Figure 1:**
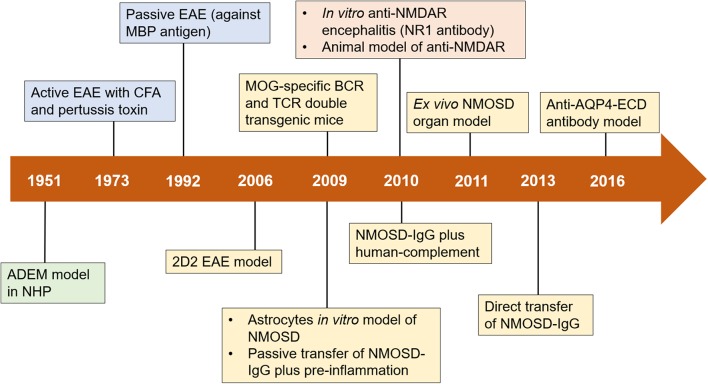
Timeline of advances in preclinical models of neuroimmune diseases. Important milestones in the development of preclinical models are shown in green boxes (for ADEM), blue boxes (for MS), yellow boxes (for NMOSD), and orange boxes (for anti-NMDAR encephalitis). ADEM, acute disseminated encephalomyelitis; AQP4, aquaporin-4; BCR, B cell receptor; CFA, complete Freund's adjuvant; EAE, experimental autoimmune encephalomyelitis; ECD, extracellular domains; MBP, myelin basic protein; MOG, myelin oligodendrocyte glycoprotein; NHP, nonhuman primate; NMDAR, N-methyl-D-aspartate receptor; NMOSD, neuromyelitis optica spectrum disorders; TCR, T cell receptor; IgG, immunoglobulin G.

Immune-mediated inflammatory diseases of the central nervous system (CNS) are a group of neurological disorders in which inflammation and/or demyelination are induced by cellular and humoral immune responses specific to CNS antigens. They include diseases such as multiple sclerosis (MS), neuromyelitis optica spectrum disorders (NMOSD), acute disseminated encephalomyelitis (ADEM) and anti-NMDA receptor encephalitis (NMDAR encephalitis). They are developed mainly through self-reactive cellular and humoral immune responses against CNS tissue antigens, such as glial and neuronal proteins (1, 2). Patients may develop a variety of neurological signs and symptoms such as sensitive and motor deficits, ataxia, visual impairment, behavioral changes and memory loss, according to the affected CNS region and target antigen (3). In the last decade, many pathophysiological aspects of the neuro-immunological disorders have been reported based on experimental models. Through these models, it is clear that autoantibodies against aquaporin-4 (AQP4) IgG are highly pathogenic and promote astrocyte injury (4, 5). Other models showed that antibodies against the GluN1 subunit of the N-methyl-D-aspartate receptor (NMDAR) lead to neuronal dysfunction and modulate receptor expression in hippocampal neurons. These findings explain the memory deficit and behavioral changes seen in patients with NMDAR encephalitis (6–8). The experimental autoimmune encephalomyelitis (EAE) model used in the MS research demonstrated that T and B cells are involved on the inflammatory response, neurodegeneration and demyelination (9–13). Moreover, recombinant antibodies against the myelin oligodendrocyte glycoprotein (rhMOG), in rhesus monkeys are able to induce ADEM and reproduce the main clinical symptoms of human disease in a genetically similar model (14–16). In this context, the pre-clinical models (i.e., *in silico, in vitro, in vivo*) have been showed as an important tool to understand the molecular and cellular mechanisms underlying each disorder (Figure 1). Here, we review the most relevant pre-clinical models in the neuroimmunology area and relate them to the clinical practice. We also explain the purpose and limitation of each one, as well as the recent advances in this field.

## Multiple Sclerosis (MS)

MS is a chronic inflammatory demyelinating disease of the CNS that compromises neuronal axons and causes myelin sheath damage, being responsible for neurological disability in young adults (17). In addition, MS is the most common non-traumatic cause of wheelchair use among those aged 18–64 years, and the third most common cause of paralysis, afflicting ~2.5 million people worldwide (18).

MS affects 3–4 times more women than men, especially Caucasian individuals. Nonetheless, its etiology remains unknown, even with the identification of risk factors (i.e., genetic susceptibility and environmental factors including vitamin D, Epstein-Barr virus infection and obesity in youth) (19–21). MS patients usually show sensory, motor and/or visual impairment due to demyelinating CNS lesions (22–24). Furthermore, the disease may present different clinical forms, being classified as: relapsing-remitting MS, secondary progressive MS and primary progressive MS. Approximately 85–90% of patients have relapsing-remitting MS. After 10 years from the disease onset, patients with relapsing-remitting MS may evolve to secondary progressive MS. The primary progressive MS is the most distinguishable form, accounting for 10–15% of the cases of MS (25).

### Pathophysiology of MS

The demyelinating CNS lesions are a hallmark of MS, which is characterized by immune cell infiltration across the blood-brain barrier (BBB), promoting inflammation, myelin injury, gliosis (i.e., activation and proliferation of glial cells) and axonal disruption (26). Early MS lesions have shown a variety of immune cells, including macrophages, CD8^+^ T cells, whereas low numbers of CD4^+^ T cells, B cells and plasma cells. During the disease course, it is common to observeiffuse inflammatory T and B cells, followed by microglia and astrocyte activation. Consequently, more pronounced gray and white matter atrophy is seen in the chronic phase, whereas microglia and macrophages remain activated throughout the disease course (27).

Regarding the mechanisms underlying immune dysregulation, it has been showed that the antigen-presenting cells (APCs) (e.g., dendritic cells) have a key role to communicate with naïve CD4^+^ T cells and shape the adaptative immune response. Posteriorly, these cells differentiate into interferon gamma-secreting (IFN-γ) Th1 cells through the presence of interleukin (IL)-12. On the other hand, the IL-23 cytokine modulates naïve CD4^+^ T cells into IL-17-secreting Th17 cells. Together, these pro-inflammatory cells have been observed within the brain and active demyelinating plaques in MS patients (28).

Additionally, the components of the myelin sheath, such as myelin basic protein (MBP), proteolipid protein and myelin oligodendrocyte glycoprotein (MOG) have been recognized as an autoantigen in MS patients, mainly by circulating CD4^+^ T cells. In contrast, healthy people also have been shown this immune profile, leading to questions about the limitation of techniques in detecting anti-myelin peptides (29).

### MS Clinical Symptoms

The clinical symptoms of MS are diverse and may result from impairment of neuronal pathways. Optic neuritis is the first neurological signal in ~25% of MS patients, which is characterized by eye pain with vision loss (30). About 43% of the patients present with myelitis and/or brainstem lesions as their first clinical attack with sensory, motor, and autonomic dysfunction (31).

Motor impairments affect about 30–40% of individual with MS and related disability may increase during the disease course. Pyramidal signs are typically observed, including pronounced reflex and clonus, as well as paresis and spasticity. Moreover, brainstem and cerebellar symptoms are common such as nystagmus, diplopia, ataxia and gait imbalance, dysmetria, slurred speech and dysphagia (32).

Other symptoms can also be developed such as cognitive impairment, affective disorders, fatigue, sleep disorders, bladder and bowel dysfunction, and sexual dysfunction (33, 34).

### Overview of MS Animal Models

The most widely used experimental model for the study of MS is the experimental autoimmune encephalomyelitis (EAE). EAE can be induced in different animal species, but rodents are the best model to understand the autoimmunity and inflammation-evoked neurodegeneration mechanisms (35). For example, natalizumab, a monoclonal antibody that inhibits α4β1 integrin resulting in the reduction of leukocyte adhesion and diapedesis at BBB, has been initially developed in EAE experiments (36).

It is important to say that, depending on the scientific question, many key aspects have to be analyzed when translating EAE data to MS, such as: (i) disease induction—the use of adjuvants (e.g., CFA and Pertussis toxin) is critical for chronic models; (ii) disease course—the mouse strain interfere in the course of the disease, for example, in SJL/J mice could be developed relapsing-remitting form, whereas the progressive state in C3HeB strain; (iii) CNS damage—EAE models usually affects the spinal cord, whereas in MS patients the inflammation occurs frequently in the brain; (iv) immune cell infiltration, usually CD4^+^ T cell is predominant in EAE model, on the other hand, CD8^+^ T cell responses dominate in human pathology (37). All of these features must be considered during the experimental study design.

There are two main different approaches to EAE induction: (i) active immunization through myelin peptides; or (ii) passive or adoptive EAE by the transfer of encephalitogenic T cells (38). Firstly, active immunization can be displayed in susceptible rodent strains (e.g., mice, rats, guinea pigs) or non-human primates (NHP) through subcutaneous administration of encephalitogenic antigens including myelin basic protein (MBP), proteolipid protein (PLP), myelin-associated glycoprotein (MAG), as well as myelin oligodendrocyte glycoprotein (MOG) (22, 39). Complete Freund's adjuvant (CFA) is the most used adjuvant to elicit EAE, although it generates granulomas at the inoculation site and lesions (40). Importantly, strain, age, sex, proportion of encephalitogenic peptide and adjuvant are crucial for EAE development (37). Secondly, EAE can be passively evoked through the transfer of MBP-specific CD4^+^ T cells by inoculation into naïve recipient animals (41). This passive transfer model is valuable to assess mechanisms controlling immune surveillance, effector phase of disease and T-cell-mediated neuroinflammation (11). Moreover, T and B cell manipulation before transfer, with stimulation of different cytokines and chemokines, enables the study of different subtypes of T and B cell implicated in EAE.

### Immune and Non-immune Signaling in the EAE

EAE demonstrates various histopathological and immunological particularities. The CD4^+^ T cells are activated in the lymph node and spleen. Subsequently, the cells leave the efferent lymphatic vessels, migrate to the circulation and acquire the ability to produce different pro-inflammatory cytokines (e.g., tumor necrosis factor-α–TNF-α, IFN-γ, IL-17, granulocyte-macrophage colony-stimulating factor—GM-CSF) and increase the expression of selectins, integrins (e.g., very late antigen-4—VLA-4 and lymphocyte function-associated antigen 1—LFA-1) and other adhesion molecules on their surface to reach the cerebral parenchyma (42).

Th1 and Th17 cells are the main CD4^+^ T cell subsets implicated in the model. Additionally, studies have shown that Th17 cells from mice have different functions when compared to humans (43). Especially in the EAE model, Th17 cells contributes to GM-CSF secretion and consequently induces chronic inflammation, whereas Th1 cells and other cell subsets are the primary source of this cytokine in humans (44). Ustekinumab, a monoclonal antibody that modulates IL12 and IL-23 involved in Th1 and Th17 cell differentiation, has been investigated partly due to EAE studies (45). In MOG-elicited EAE, Th17 cells were shown to mediate axonal damage without the participation of T cell receptor (TCR) (10). Also, CD8^+^ T cell-modulated cytotoxicity may contribute directly to axonal damage, although electrically active neurons do not typically express major histocompatibility complex (MHC) class I proteins. Importantly, inflammatory white matter demyelination is not adequately reproduced in the brain, whereas it is an important histopathological feature of MS (12).

Accumulated evidence suggests that B cells play an important role in MS immunopathology. A few year ago, Matsushita and colleagues showed that mice lacking CD19—a marker of B cell—increase the severity and delay the recovery of EAE (9). Regulatory B cell-deficient mice fail to recover and develop chronic paralysis (46). These results indicate that some B cells such as regulatory B cells may be crucial for the resolution of inflammation and EAE. In contrast, B cell depletion after EAE induction reduces clinical scores compared to controls, apparently through reduction of CNS-penetrating, autoantigen-specific CD4^+^ T cells (47). More recently, Kumari and coworkers demonstrated that demyelination in the cervical region correlated with the infiltration of CD19^+^ B cells in the EAE model (13). In summary, depending on the B cell population balance (autoreactive vs. regulatory), the responses may be completely different in the EAE model.

In EAE model, the inflammatory response is accompanied by activation of microglia and astrocytes, leading to marked axonal damage and demyelination, mainly seen at the peak of the disease (12). Astrocytes are crucial to maintaining BBB homeostasis. At the onset of clinical motor deficits in EAE, reactive astrocytes show the following features: (i) proliferation; (ii) extensive hypertrophy of cell body; and (iii) elongation of fibrous branches (48). As a consequence of this chronic process, oligodendrocyte death and axonal injury may occur in the EAE model in some susceptible strains (49).

### EAE Clinical Signs

The spinal cord is the most affected region in the EAE, while only sparse inflammation is observed in the brain, justifying the motor impairment after EAE onset (37). Regarding motor symptoms, EAE are usually scored from grade 0 (normal) to 5 (moribund), according to a functional deficit characterized by ascending paralysis, starting at the loss of tail tone (grade 1), hind limb paresis (grade 2) and paralysis (grade 3), followed by progression to the upper limbs (grade 4) (50).

EAE has also been used as an *in vivo* model for validating symptomatic treatments. Baker et al. demonstrated that the treatment with cannabinoids was able to control spasticity and tremor in EAE (51). Furthermore, bladder signs of MS could be mimicked in EAE and the utility of future drugs for neurogenic bladder impairment in MS could be tested in this model (52). More recently, Silva et al. (53) showed that calcium channel blockage modulates a variety of symptoms related to the EAE model, such as physical and thermal pain, neurological score, motor coordination and memory (53).

### Limitations of EAE

The EAE model has contributed significantly to the understanding of autoimmunity and neuroinflammation in MS, allowing the development of novel therapeutic approaches for the disease. Nonetheless, this model has some limitations regarding the pathogenesis of human MS: (i) EAE provides limited information about MS progression because most *in vivo* models consist of the monophasic phenotype; (ii) C57BL/6 mice are not suitable for the study of progressive MS; (iii) remyelination is difficult to be studied in EAE because limited information is available; (iv) therapeutic approaches with neuronal growth and survival factors have been unsatisfactory; and (v) EAE mainly affects spinal cord white matter (54).

## Neuromyelitis Optica Spectrum Disorder (NMOSD)

NMOSD is an immune-mediated inflammatory CNS disorder with severe attacks of optic neuritis and transverse myelitis. Historically, NMOSD was considered a variant of MS, but since the discovery of serum antibodies against aquaporin-4 (AQP4-IgG) (55), it has been clearly considered a distinct entity (56, 57).

The NMOSD lesions predominantly affects the optic nerves, area postrema and spinal cord (21). Tissue damage is usually severe with a high risk of permanent disability such as blindness, severe sensory-motor deficits, paralysis and death (58, 59). Optic neuritis (ON) in NMOSD may be unilateral or bilateral, compromising visual and spatial ability, color sensitivity and pupil function (58). The great majority of ON attacks are painful and worsened by ocular movement (60, 61). ON lesions are extensive, affecting the entire length of the nerve from the orbit to the optic chiasm (61). Patients with ON have thinning of the retinal nerve fiber layer and loss of the ganglionic layer. These changes are often observed in NMOSD patients, but may also appear in MS and other inflammatory neuropathies (62). In the spinal cord, NMOSD lesions are usually extensive (more than three segments on the sagittal view) and located in the central portion on the axial view (61). When the area postrema is affected, the patients present persistent nausea, vomiting (>48 h) and intractable hiccups (60).

NMOSD has a prevalence of 1–8 cases per 100,000 individuals. Similar to other autoimmune pathologies, predominant in the female population (8:1). Although the common age at disease onset is between 30 and 40 years old, the disease can also occur in children and the elderly. It is more prevalent in non-Caucasians (57, 60, 61).

### Pathogenesis of NMOSD

AQP4-IgG is produced by autoreactive B cell lines. These cells secrete AQP4-IgG after IL-6 stimulation in association with CD4^+^ T cells and Th17. AQP4-IgG antibodies are of the IgG1 subtype, so they are dependent on T-B cell interactions. As infiltrating T cells are detected in typical NMOSD lesions, they may be responsible for BBB disruption and facilitate the entrance of AQP4-IgG in the CNS, as well as other inflammatory cells such as granulocytes and macrophages (63–66).

AQP4-IgG antibodies enter the CNS by endothelial transcytosis or through areas such as circumventricular regions (67). The binding of AQP4-IgG antibodies to AQP4 downregulates the protein on the surface of the astrocytic membrane, disrupting water homeostasis in the CNS (65). Moreover, *in-vitro* and *in-vivo* experimental models have shown that AQP4-IgG promotes an inflammatory response in astrocytes, increases BBB permeability and activates the complement system by the classic route (C1q). Furthermore, induces complement-dependent cytotoxicity (CDC) through the deposition of activated complement proteins and antibody-dependent cell cytotoxicity (ADCC) by the activity of natural killer (NK) cells (67).

Complement activation induces the production of anaphylatoxins that attract inflammatory cells, such as monocytes and granulocytes, into the CNS (60, 68). These inflammatory processes contribute to the formation of classic lesions, characterized by astrocytic injury, loss of AQP4 immunoreactivity and glial fibrillary acidic protein (GFAP), IgG and activated complement deposition and inflammation around blood vessels. This inflammatory response is amplified in the CNS and secondarily affects oligodendrocytes, causing damage to the myelin sheath, axons and neuronal death (60, 67, 69).

### Experimental Models of NMOSD

After identifying the AQP4-IgG antibodies and their specific target, it was expected that the development of experimental models of NMOSD would be simple. However, it was found that AQP4-IgG passive transfer to animals was not enough to reproduce the disease, requiring active induction of inflammatory response by EAE and/or human complement. Therefore, there is no experimental model of NMOSD that fully reproduces the clinical, pathological and immunological characteristics observed in humans. However, the current models reproduce some important aspects of the human disease to address key-points raised by clinicians about the importance and pathogenicity of AQP4-IgG antibodies, the participation of immune cells and cytokines to lesion formation, as well as the role of T and B cells in the development of the disease (70, 71).

#### *In vitro* Models

Early studies with cellular models showed that AQP4-IgG has deleterious action on astrocyte cultures. Kinoshita et al. (4) developed an *in vitro* NMOSD model showing the deleterious effect of AQP4-IgG on astrocytes and NMOSD pathogenesis. The study showed that AQP4-IgG in association with complement induced astrocyte death. Moreover, they demonstrated that AQP4-IgG has a deleterious action by itself and can change the phenotype and function of astrocytes (4, 72). Subsequent *in vitro* studies show that AQP4-IgG has the ability to increase BBB permeability, depolarize AQP4 in the astrocytic membrane (73), induce ADCC and CDC and consequently stimulate the inflammatory cells proliferation (74). Furthermore, the binding of AQP4-IgG to AQP4 expressed in astrocytes results in functional changes such as target internalization by endocytosis (75), decreased expression of AQP4 and modification of AQP4 function (67).

The development of these models was possible because AQP4-IgG recognizes the extracellular domains (ECDs) of AQP4 (76–78). In 2016, Hung et al. developed two monoclonal antibodies (mAbs) against the ECDs of AQP4—to establish a mouse NMOSD cellular model—using a baculoviral display method, named E5415A and E5415B. The first mAb recognized M1 and M23 isoforms, and the second mAb only recognized the square-array-formable M23 isoform. Overall, the results indicated that a large cluster of AQP4 was constructed by anti-AQP4-ECD antibody, leading to endocytosis and degradation of AQP4 by lysosomes (79).

Research based on the cell models clarified the main pathogenic mechanisms of AQP4-IgG in NMOSD. Today, we know that antibodies are the key to destructive astrocyte lesions in the presence or absence of complement. Astrocyte culture exposed to AQP4-IgG becomes a useful model for screening drugs that block AQP4 channels in pathogenic astrocytes or drugs that protect astrocytic death (57, 72).

#### Ex vivo Models

AQP4-IgG purified from patients with NMOSD can recognize and bind to extracellular AQP4 of living astrocytes from humans (80), rats (63) and mice (80). However, *in vitro* models cannot mimic the CNS tissue damage cascade. Thus, rodent spinal cord culture, optic nerve, hippocampal or cerebellar slices can be used as an *ex vivo* model to study NMOSD pathogenesis, to screen drugs or to investigate the influence of mediators in the pathogenesis of NMOSD (5, 62, 81).

Zhang et al. (5) developed an *ex vivo* model of NMOSD using spinal cord slices to evaluated the pathogenicity of AQP4-IgG and the involvement of specific inflammatory cell types and soluble factors in NMOSD lesions (5). The study showed that exposure of spinal cord slices to recombinant AQP4-IgG and complement reproduces NMOSD lesions characterized by severe loss of AQP4, GFAP and MBP. The tisse had evidence of astrocytic swelling, microglial activation, complement deposition and secondary demyelination (5, 82). This model also demonstrated that neutrophils are able to exacerbate the damage, increasing AQP4 and GFAP loss in the presence of AQP4-IgG, showing typical characteristics of the disease in humans. Macrophages and NK cells also increase the severity of human disease and *ex vivo* lesions in the presence of AQP4-IgG and complement. Soluble factors such as IL-1β, IL-6, TNF-α and IFN-γ also exacerbate the *ex vivo* lesions. It is not clear what the role of cytokines in NMOSD, but it is known that IL-1β and IL-6 are elevated in the CSF patients (5).

Another study conducted by Felix et al. (62) showed that exposure of retinal cultures to passive AQP4-IgG results in primary, complement-independent retinal pathology, which might contribute to retinal abnormalities seen in NMOSD patients. They showed that AQP4-IgG stimulates the endocytosis of AQP4, resulting in low expression of the protein on the cell membrane (62).

*Ex vivo* models allow us to simulate tissue damage, as well as to identify inflammatory mediators and soluble factors that may be involved in NMOSD. However, the model has limitations, because it does not allow investigating the participation of multi-factorial cell and soluble mediators from the periphery, nor the influence of BBB. Nevertheless, they are useful for investigating issues that cannot be studied using *in vivo* models, such as the individual role of inflammatory cell subsets and cytokines in the pathogenesis of NMOSD lesions.

#### Passive Transfer of AQP4-IgG in EAE Models

An NMOSD-like pathogenic process can be obtained from the AQP4 protein structure similarity between humans and rodents (57). Some papers have recently shown that passive transfer of AQP4-IgG from patient serum or plasma to a rat pre-immunized with MBP emulsified with CFA can evoke NMOSD-like injury (72). The NMOSD model by passive transfer showed that the AQP4-IgG antibodies bind to the astrocyte membrane with loss of AQP4 and GFAP, deposition of IgG and activated complement, granulocyte influx in perivascular areas and astrocyte injury (70, 72, 83, 84). The models developed by Kinoshita et al. (85) and Kurosawa et al. (69), aimed to reproduce the main pathological characteristics of human NMOSD in pre-immunized rats, using purified AQP4-IgG from patients given by intraperitoneal injections. In this model, the lesions resembles human pathology with massive infiltration of neutrophils, eosinophils and macrophages around blood vessels in the gray matter, perivascular deposition of IgG and complement, microglial activation and loss of immunoreactivity of AQP4 and GFAP (69, 70, 85). In addition, they once again showed the involvement of reactive T cells in the NMOSD pathology. Previous immunization with MBP/CFA (EAE) is required to induce infiltration of reactive T cells into the brain parenchyma, which promotes a pro-inflammatory environment and increase BBB permeability, thus allowing the entry of AQP4-IgG into the CNS (70, 83, 84, 86).

However, this NMOSD/EAE also has limitations. Firstly, it requires a large amount of AQP4-IgG for a single NMOSD/EAE animal injection. Secondly, in Lewis rats, Th1 cells are responsible for CNS disease, whereas NMOSD in humans may also have a Th17 cell response. Although mice produce a Th17 cell response, they cannot be used to evoke NMOSD/EAE because human AQP4-IgG is unable to activate murine complement (70, 87). Third, in this model it is difficult to evaluated demyelination, when myelin injury occur, is secondary to astrocyte depletion and form after a prolonged period of AQP4-IgG infusion (86). In humans, antibodies are produced continuously, serial AQP4-IgG injections may be required to simulate long term exposure to pathogenic antibodies.

#### Intracerebral AQP4-IgG Direct Injection

In this model, AQP4-IgG derived from NMOSD patients is injected directly in the brain or ventricular system of rodents (70, 71, 83). Saadoun et al. (88) injected AQP4-IgG supplemented with human complement directly to the cerebral hemisphere of mice. The model was successful in reproducing histological characteristics of human NMOSD. The first lesions occurred just 12 h after the injections and include loss of AQP4 and GFAP, glial cell edema, myelin damage and early axonal injury. After 1 week, they observed an extensive inflammation in the right hemisphere and perivascular inflammation within 1 mm of the needle tract, perivascular complement deposition, infiltration of mononuclear and polymorphonuclear cells and extensive demyelination with neuronal cell death in the injected hemisphere. The study also showed that many astrocytes around the lesions had a reactive phenotype, characterized by high GFAP expression and changes in their morphology (88).

Based on the same method but in rats, Asavapanumas et al. (89) applied a single intracerebral injection of AQP4-IgG without human complement supplementation in naïve adult rats, because human AQP4-IgG is able to activate the classical complement pathway of rats. This model reproduced robust lesions in the animals 5 days after immunization. The lesions presented loss of AQP4, GFAP and myelin, perivascular deposition of activated complement, BBB disruption evidenced by albumin extravasation, infiltration of granulocytes and macrophages, microglial activation and neuronal degeneration (89). The lesions are similar to the model described by Saadoun et al. (88), but here they were generated only with AQP4-IgG, without previous neuroinflammation or complement administration, since this model uses the rat endogenous complement. In this method, there is no administration of other components in the rat brain that can influence the formation of the lesions.

The main advantage of these models is that it requires small amounts of purified AQP4-IgG to be executed, thus these models are an excellent tool for the study of new drugs and small molecules that can inhibit AQP4-IgG binding to its target. Furthermore, as these models are reproduced in both rats and mice, studies can use the large repertoire of transgenic or knockout rodents currently available, which may provide us further insight into the role of individual molecules in lesion formation. In addition, inflammation and tissue destruction are quantifiable, allowing to measure the local effect of new therapies (70, 88).

However, the models have disadvantages. In the mice model, it is necessary a co-administration of human complement since AQP4-IgG *per se* is not able to activate the mice complement system. In addition, the target tissue is constantly manipulated due to brain injections can alter the susceptibility of the CNS to react to additional inflammatory stimuli (70). Lastly, this model does not address the other immune mechanisms involved in the AQP4-IgG production or its access to the CNS. To evaluate these aspects, models based on peripheral administration of AQP4-IgG are more suitable.

#### Intrathecal AQP4-IgG Direct Injection

Chronic intrathecal infusion of AQP4-IgG or recombinant human anti-AQP4 antibodies using implanted catheters (90, 91) leads to a NMOSD-like pathology primarily in the spinal cord and optic nerve (92). Geis et al. (91) investigated the intrinsic effects of AQP4-IgG by inducing a chronic animal model of NMOSD through repeated intrathecal injections of recombinant AQP4-IgG into the spinal cord of rats. The model caused progressive and reversible spinal cord pathology independent of complement, with marked intraspinal IgG deposition, loss of AQP4 immunoreactivity and astrogliosis in the region adjacent to the implanted catheter, but with preservation of astrocytes, axons, myelin and oligodendrocytes. It was also possible to note a mild intraspinal infiltration with macrophages restricted to the area adjacent to the tip of the catheter, as well as reduced expression of the glutamate transporter (GLT-1) (91), proposed to contribute to NMOSD pathophysiology (93). Mild to moderate progressive myelopathic signs were also observed in this model, starting with unilateral paresis later evolving to an asymmetric paraparesis of the hind limbs. There were no deficits in the forelimbs, indicating that the pathology was restricted to the thoraco-lumbar level (91).

In a refined model, Marignier et al. (92) showed that the prolonged infusion of AQP4-IgG directly into the rats' CSF, leads to the diffusion of antibodies in the CNS, affecting the optic nerves and spinal cord, structures relevant to human pathology. The study showed the ability of AQP4-IgG to induce morphological and functional changes in astrocytes, modify myelin structure, destroy oligodendrocytes and axons, compromising the motricity of animals. The lesions had deposition of IgG, reduced expression of AQP4, loss of myelin and axons. Moreover, reduced expression of the glutamate transporters (GLAST and GLT-1) was also observed in the lesions, reflecting the reduction of glutamate uptake by astrocytes. However, the inflammatory process was mild with few cellular infiltrates in the CNS. There was no microglial activation or deposition of activated complement components, typical characteristics of human disease that were not reproducible here (92).

Intrathecal infusion models allow evaluating the AQP4-IgG antibodies action *in vivo* at relevant tissues such as spinal cord and optic nerves, independent of additional effector mechanisms. Also, they may reproduce clinical characteristics similar to those of humans, such as walking deficits, paresis, and paraparesis. However, the model does not show the main pathophysiological characteristics of human NMOSD, the inflammation and massive infiltration of inflammatory cells in the CNS and the participation of the complement system in the formation of the lesion. Despite this, intrathecal infusion models showed that AQP4-IgG alone is able to induce astrocytopathy, independent of complement and neuroinflammatory processes, leading to demyelination and axonal damage. In addition, they also showed that AQP4-IgG interferes in glutamate homeostasis. Therefore, these models can be useful to investigate whether glutamatergic excitotoxicity contributes to NMOSD pathophysiology.

## Acute Disseminated Encephalomyelitis (ADEM)

ADEM is an acute inflammatory demyelinating CNS disorder mainly affecting the brain white matter and the spinal cord. It is usually monophasic and may affects individuals of all ages but it occurs more frequently in children and young adults males (94, 95). ADEM is commonly associated with recent history of infections and vaccinations, but it may occur spontaneously (96, 97). The disease has an acute onset with focal or multifocal neurologic deficits (95) associated with encephalopathy and confusional state (98). Clinical manifestations include fever, malaise, nausea, vomiting, focal and/or diffuse neurological symptoms such as headache, meningism, seizures, cranial nerve palsies, ataxia, and coma (97, 99).

ADEM etiology is still unclear. It is believed that antigens such as MBP, PLP and MOG are targets of antibodies or reactive T cells in the disease. The presence of T cells reactive to MBP was observed in the CSF of patients diagnosed with ADEM. Another study reported serum IgG antibodies reacting to various myelin proteins, but the pathogenic potential is unclear (100).

Recently, some groups have reported specific serum MOG-IgG antibodies to conformational epitopes of MOG in pediatric ADEM cases (21). These antibodies may activate the complement cascade and initiate cell death through NK cells, contributing to the pathogenesis. However, demyelination does not occur only through the action of MOG-IgG, the presence of pro-inflammatory cytokines is also necessary to induce injury (100). Pediatric MOG-IgG positive patients have a characteristic lesion pattern that can be seen on magnetic resonance imaging (MRI), demyelinating lesions are bilateral and usually large, concentrated around blood vessels and distributed throughout the parenchyma, including the cortex, thalamus, basal ganglia, spinal cord, brain stem and cerebellum (21). Demyelinating lesions are concentrated in the perivascular region and are surrounded by macrophages containing myelin remains and infiltrates of T and B cells, plasma cells and granulocytes, together with activated microglia and reactive astrocytes (97, 98). Axons are generally preserved, but eventually, show features of acute injury and the vessel walls shows fibrinous exudates that can lead to adjacent necrosis, indicating an overlap of ADEM and acute hemorrhagic leukoencephalitis (21). After treatment, most ADEM patients recover completely, but ~18% of patients remain with mild to moderate neurological deficits (100).

Due to the poor understanding of disease mechanisms involved in ADEM, experimental models are scarce, there are no *in vitro* and *ex vivo* models described, and *in vivo* models are based on the adaptation of MS/EAE protocols, as described below.

### ADEM by Active EAE in NHP Models

In addition to applying to the development of MS and NMOSD models, many EAE models have a monophasic disease that may also be used as an ADEM model. However, *in vivo* models use NHPs, such as rhesus monkeys (*Macaca mulatta*). The rhesus monkey is a primate that shares immunological characteristics with humans. Therefore, it is very useful for reproducing pathological characteristics similar to those observed in patients, as it has a rich repertoire of self-reactive T cells in the peripheral system (14).

Initially, NHP models were developed to investigate the pathological mechanisms of MS, in different monkey species: rhesus monkeys (*Macaca mulatta*), cynomolgus monkeys (*Macaca fascicularis*) and common marmosets (*Callithrix jacchus*). However, instead of manifesting clinical features and lesions similar to MS, rhesus monkeys showed severe acute lesions similar to human ADEM. The rhesus monkeys were immunized with myelin antigens such as MBP, PLP or recombinant antibodies against MOG (rhMOG) in combination with adjuvants (CFA or IFA). This immunization induces a severe and hyperacute neurological syndrome, especially when used rhMOG, which may progress to the death of the animal (14). The first clinical signs appear in only 12 h after immunization of animals, starting with weakness, hemiparesis, paresthesia and ataxia, progressing to paresis, paralysis and coma. The macroscopic and histopathological lesions of the white matter in NHP models show similarity to human ADEM, affecting cortical white matter, corpus callosum and subpial white matter regions. Spinal cord lesions may also occur but that less extent. Optic nerves and brain stem lesions are less frequent (15, 101). Lesions in NHP models are severe, usually containing necrotic and hemorrhagic areas, and generalized inflammation with massive neutrophils infiltration, perivascular demyelination, loss of oligodendrocytes and axonal damage, pathological characteristics that are also observed in patients with ADEM (15, 16, 101).

For a long time, EAE murine models (C57BL/6 or SJL/J) prevailed in studies of autoimmune inflammatory diseases. These models were essential for establishing general concepts of inflammatory pathologies, as well as the immune mechanism activated by autoantibodies. Conversely, rodent models have limitations to reproduce the pathophysiology of neuroinflammatory diseases. In addition, rodents used as models are specific pathogen-free (SPF); thus, their immune system is not modulated by pathogenic action and environmental factors, as occurs with humans (15, 101).

To approximate the experimental models to human diseases, the NHP models were created. These models are extremely useful as preclinical models of autoimmune inflammatory diseases because of their genetic and immunological similarity with humans, acquired during the evolution of the species. NHPs also share common neuro-anatomical structures with humans (14, 16), which allows identifying the onset of the lesions, their progression or regression, especially through imaging techniques such as MRI. Thus, these models refined our understanding of the pathophysiology of ADEM, filling the gaps from rodent models and translating this information to human disease (101).

### Implication of NHP Models

EAE can be induced in different species of primates. However, clinical manifestations differ among species. EAE in marmosets follows a course similar to MS, but in rhesus monkeys it promotes a more acute and aggressive response, resembling ADEM (16). This is due to the influence of the immunizing antigen and the injected adjuvants. These are important factors for EAE induction, which can directly interfere in the severity of disease (101).

The CFA and IFA adjuvants are crucial elements for EAE induction, modulating the course and severity of the disease. Rhesus monkeys immunized with CFA develop severe ulcerative granulomas on the skin, at the site of application, which causes discomfort and pain to the animal. For this reason, the use of CFA for immunization is considered unethical. To soften the side effects, CFA was replaced with IFA, which does not contain *Mycobacterium* strains but maintains the same EAE severity observed in CFA immunization. In contrast, the epidermal lesions in the animals are lighter, characterized by discrete granulomatous dermatitis without ulceration (15, 101).

Even in NHP models reproducing characteristics of human ADEM, including demyelination and injury to axons, symptoms such as pain, depression and cognitive deficits cannot be evaluated because of the severity of the disease and short clinical course. So far, there are no standardized methods to evaluate sensory and cognitive impairment in these animal models (101).

## Anti-NMDA Receptor Encephalitis (NMDAR Encephalitis)

Autoimmune encephalitis comprises a group of disorders in which autoantibodies are produced against synaptic antigens and neuronal surface proteins, leading to brain dysfunction (102, 103). Clinical manifestations include prodromal symptoms such as fever, headache, nausea and vomiting, progressing to neuropsychiatric manifestations, behavioral changes, memory deficit, psychosis, autonomic instability, seizures and coma (102–110). There are two major groups of autoimmune encephalitis: those that produce antibodies against intracellular antigens and those that produce antibodies against neuronal surface antigens (111), such as encephalitis against NMDA, GABAb, AMPA and CASPR2 receptors (112–115). NMDAR encephalitis is the most common and most studied in experimental models (8, 116).

NMDAR encephalitis affects children and adults with female predominance (prevalence of 3–5 cases per 1,000,000 individuals) (60). The disease is associated with tumors such as ovarian teratoma, but may appear spontaneously (116). Clinical manifestations include psychiatric symptoms such as confusion, abnormal behavior, paranoia and hallucinations, other symptoms such as memory deficits, seizures, dyskinesia, autonomic instability, catatonia, hypoventilation, lethargy and language deficits may also appear (8, 60). After treatment, 75% of patients have a substantial clinical recovery, which occurs in reverse order to the development of symptoms, accompanied by a decline in antibody titers (117).

NMDAR encephalitis is characterized by production of IgG1 antibodies against the amino-terminal domain of the ionotropic glutamate receptor (NMDA) subunit GluN1 (60, 116). The NMDA receptor is a heterotetrameric ion channel expressed at the postsynaptic terminal of neurons throughout the CNS. In the cortex and hippocampus, NMDA receptors are composed of one GluN1 subunit and two GluN2 subunits, called GluN2A and GluN2B (118, 119). This receptor is essential to promote synaptogenesis, synaptic plasticity (mainly related to memory) and neuronal signaling (60, 120). Therefore, an impairment of its functions may lead to functional and structural changes in the brain. The GluN1-IgG antibodies produced in NMDAR encephalitis induce the receptor internalization across the membrane, causing a redistribution of NMDA receptors on the neuronal surface (120, 121), resulting in reduced nervous signal conduction, memory and behavioral changes due to decreased receptor density in the hippocampus (60). Due to its specificity, the GluN1-IgG antibodies do not change the location and expression of other synaptic proteins, as well as the number of synapses, dendritic spines and cell survival (6, 122).

In the last years, many *in vitro* and *in vivo* studies have been using passive or active immunization to show the pathogenic potential of specific antibodies. Also, it provides a basis to study new therapies, as we will present below.

### *In vitro* Model

In these models, cultures of hippocampal neurons are exposed to antibodies against the NMDA receptor GluN1 subunit (GluN1-IgG), derived from the CSF of patients with NMDAR encephalitis. Studies have shown that GluN1-IgG antibodies reduce the level of NMDA receptors on the surface of the neuron (6, 123), It can reduce the response of neurons to glutamate, impairing essential neuronal functions dependent on glutamatergic signaling (117). The studies also observed that this change is dependent on antibody titers and that its effects may vary with the change in titers during the course of the disease, suggesting a dose-dependent effect (6). The synaptic location of NMDA clusters has also decreased dramatically; however, when removing GluN1-IgG antibodies from cultures, the NMDA clusters density returned to baseline levels (6, 123).

Although GluN1-IgG antibodies reduce NMDA receptor density on the neuronal surface due to its internalization, they do not interfere in the synapses nor modulate postsynaptic receptor density such as PSD-95, GluR1, GluR2, GABA, or AMPA. Moreover, they not compromise excitatory neuron structures or their viability. These results indicate that GluN1-IgG antibodies specifically target the NMDA receptor changing its functionality, but they have no deleterious effects on other receptors or synaptic proteins (6, 123).

The *in vitro* models helped to demonstrate how GluN1-IgG antibodies lead to neuronal dysfunction, and their ability to modulate the expression of the NMDA receptor on the neuronal surface without affecting other synaptic receptors and proteins. However, the antibodies *in vitro* effect do not necessarily reflect the pathogenic role *in vivo* and vice versa (8). These models are important tools to discover new therapies that modulate GluN1-IgG binding at NMDA receptor, induce balance of antagonistic neurotransmitters, and restore the neural network (60).

### Passive Transfer of GluN1-IgG

In these models, the GluN1-IgG is directly delivered by intraventricular administration to the hippocampal and cortex structures in C57BL/6 mice. Studies have shown that osmotic pump intraventricular infusion for 14 days using CSF of NMDAR patients in mice compromises rodent memory, alters their behavior and causes depression, similar to human NMDAR encephalitis (7, 124). Besides, there is a decrease in NMDA receptors density on the neuronal surface, especially in the hippocampus, which explains the memory impairment (7, 104). However, locomotor changes, anxiety signs and, aggressive behavior seen in patients with NMDAR encephalitis were not observed in mice (7, 124).

Another study showed an increase in neuronal excitability and, extracellular glutamate levels in the premotor cortex of rats infused with CSF patients and purified GluN1-IgG. These results suggest that GluN1-IgG antibodies are capable to interfere in glutamatergic synapses, which may induce a hyperglutamatergic state in the brain of patients and cause an imbalance in NMDA and AMPA receptors (125). Other groups have shown that GluN1-IgG antibodies significantly reduce NMDA-mediated excitatory postsynaptic potentials (EPSPs) and long-term potentiation (LTP) in the CA1 and dentate gyrus of animals exposed to the patient's CSF diagnosed with NMDAR encephalitis (126, 127).

Using the same principle of intraventricular infusion, Taraschenko et al. (128) showed that anti-NR1 antibodies are also capable of inducing seizures. Electroencephalography (EEG) showed that prolonged CSF infusion induced seizures in 10 of 11 animals. These animals had a total of 39 seizures during the 14 days of infusion, and 5% were characterized by myoclonic reflexes. In addition to causing behavioral and memory changes, anti-NMDAR encephalitis antibodies can induce seizures spontaneously, similar to those that occur in human encephalitis (128).

In this context, the *in vivo* NMDAR encephalitis models support the mechanisms proposed by *in vitro* models concerning NMDAR encephalitis pathogenesis such as cross-linking, internalization and the alteration of NMDA receptor expression on the neuronal surface. They reproduced the main clinical symptoms of human NMDAR encephalitis as memory deficits, depressive-like behaviors, and seizures, and showed that these symptoms can be neutralized by ephrin-B2 administration (the ligand of EphB2 receptor) (119, 129). Those GluN1-IgG antibodies promote a hyperglutamatergic state in the brain of animals, suggesting that excitotoxicity acts on the pathophysiology of human NMDAR encephalitis (125). Nevertheless, these models did not show movement disorders, long-term cognitive deficits or hippocampal damage observed in patients. This may be due to a limitation of the species or some inflammatory change cannot be reproduced by passive transfer models (8).

### Active Immunization With Proteoliposomes

Although passive transfer models provide useful information about the role of GluN1-IgG antibodies in NMDAR encephalitis, they do not reproduce all the disease clinical spectrum. It is difficult to determine the immune factors involved in the pathogenesis, as well as the role of inflammatory cells. Based on this, research groups have developed active immunization methods to fill these gaps. In a recent study, the mice were immunized, subcutaneously, with extracellular peptides against NMDA receptor subunit GluN1. These animals did not show behavioral changes, even in the presence of high titers of antibodies. However, psychotic behavior has been observed in animals that present a disrupted BBB. The histopathological features such as lymphocyte infiltration and activated microglia were not seen (130). However, active immunization (subcutaneously) of C57BL/6 mice with purified tetrameric GluN1/GluN2B NMDA receptors, fully assembled in liposomes (NMDA receptor proteoliposomes), induced a fulminant encephalitis phenotype within 4 weeks in mice. Clinical symptoms included hyperactivity in 86% of the animals, tight circling (50%), seizures (21%), hunched back/lethargy (11%). Immunohistological analysis showed immunoreactivity for GFAP and Iba1 in the hippocampus of mice, 6 weeks after treatment. An infiltration of immune cells such as activated macrophages, plasma cells, CD4^+^ T cells, and B cells, was also observed mainly in the hippocampus, striatum, thalamus, amygdala and neocortex of treated mice. Furthermore, all animals immunized with proteoliposomes produced GluN1-IgG and GluN2B-IgG antibodies in 6 weeks after immunization, suggesting the occurrence of a polyclonal response after disease onset. However, the response to the GluN1 epitope was predominant (118). Unlike human antibodies, mouse antibodies reacted with linear NMDAR epitopes and not limited to the amino-terminal domain of GluN1. Despite this, active immunization may be a useful approach to study new treatments (8).

Active immunization has shown great potential as an alternative to existing animal models for NMDAR encephalitis. It was evidenced that the peptide alone was not sufficient to develop the symptoms normally found in patients with encephalitis, but it was enough to cause disruption of BBB in the mice model. However, when used conformationally-stabilized holoproteins, the results obtained are much more satisfactory, with clinical symptoms resembling the NMDAR encephalitis in humans. Unlike passive transfer models, active immunization leads to a fulminant pathology, covering both clinical and histopathological aspects of NMDAR encephalitis. Therefore, it is a model that allows us to evaluate the severity of the disease course, the role of specific immune components and the potential for new therapies.

### Limitations of NMDAR Encephalitis Models

Unlike the other diseases and methods addressed in this review, in which antibody action results in typical tissue changes, the cellular and structural changes are difficult to detect in the infusion model. Moreover, animals should be exposed to CSF at elevated antibody concentrations over a long period, but the consequences of this long exposure to the animal are unknown (7). Although the active immunization models obtained promising results, it was observed that only the GluN1 peptides were not sufficient to mimic the clinic symptoms. It is observed that an entire immune cascade is necessary for the development and detection of clinical features observed in human NMDAR encephalitis.

**Figure 2:**
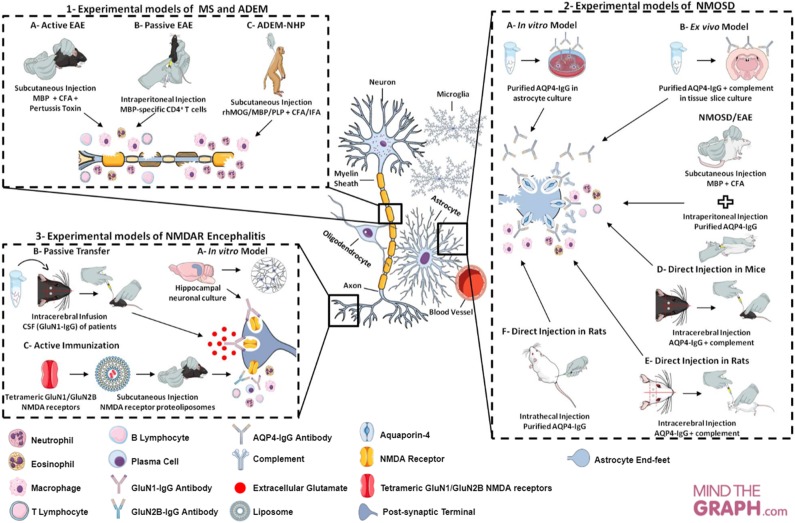
Cellular and molecular targets of neuroimmunological models. (1) Experimental models of MS in mice and ADEM in rhesus monkeys. In the MS model—called EAE—, there are two main approaches to induction: (A) active immunization through myelin antigens; (B) adoptive/passive transfer of encephalitogenic T cells. The neuroinflammatory response is characterized by cell infiltration (e.g., macrophage, neutrophil, T cell, B cell) and secretion of inflammatory mediators (e.g., cytokines and ROS). This model may mimic the relapsing-remitting or progressive phase, depending on the experimental protocol. EAE can be evoked in rhesus monkey (*Macaca mulatta*) and enables an acute and aggressive response, resembling ADEM (model C). The main features of ADEM-NHP are neutrophil infiltration, loss of oligodendrocytes and axonal damage. (2) Experimental models of NMOSD in astrocyte culture, tissue slice culture and rodents. The experimental models of NMOSD are divided into three categories: *in vitro, ex vivo* and *in vivo*. To establish *in vitro* (A) and *ex vivo* (B) models, purified AQP4-IgG is exposed to astrocyte and brain tissue slice cultures. IgG binding to AQP4 generates cytotoxicity, inflammatory response, astrocyte damage and AQP4 endocytosis, as well as complement deposition and demyelination in tissue slice culture. *In vivo* NMOSD models have two approaches to induction: (C) passive immunization through EAE induction (with myelin antigens) and subsequent purified AQP4-IgG injection; (D,E) direct immunization through intracerebral injection of AQP4-IgG and human complement; (F) or intrathecal injection of AQP4-IgG without complement. The inflammatory response is characterized by recruitment and inflammatory cell infiltration (e.g., macrophages and granulocytes), astrocytic damage, deposition of IgG and complement and loss of AQP4 and GFAP in passive immunization, as well as, astrogliosis, extensive demyelination, axonal injury and neural cell death in direct immunization. (3) Experimental models of NMDAR encephalitis in neuronal culture and mice. Hippocampal neuronal cultures are exposed to GluN1-IgG antibodies from CSF of patients with encephalitis, leading to a reduction in the expression of receptors on the neuronal surface (internalization) and decrease in synaptic currents (model A). The mouse models have two approaches to induction: (B) passive transfer of the GluN1-IgG through continuous CSF infusion directly into the cerebral hemisphere of the animals; (C) active immunization using conformationally-stabilized holoproteins. In the passive model, mice present loss of NMDA receptor expression on the neuronal surface, a decrease in synaptic currents and, consequently, memory impairment, behavioral changes and spontaneous seizures. The inflammation in active models is characterized by leukocytes infiltration, activated macrophages, plasma cells and T and B cells.

All experimental models (*in vitro, ex vivo* and *in vivo*) of CNS neuroimmunological diseases, as well as routes of administration, induction techniques and immunogens (such as proteins and peptides, purified or recombinant antibodies injected) discussed here, are represented in Figure 2 and summarized in Table 1.

**Table 1:** *In vitro, ex vivo*, and *in vivo* models of neuroimmunological disorders.

**Disease**	**Induction mechanism**	**Species/strain**	**Encephalitogenic agent**	**Pathological features**	**Advantages**	**Disadvantages/limitations**
MS	Active EAE	Rodents NHP	MBP, PLP, MAG, MOG emulsified in CFA or IFA	BBB disruption, inflammatory cell infiltration, axonal damage, demyelination	Reproduces histopathological and immunological characteristics common to human MS	Generates granulomas at the inoculation site and lesions, leading to pain symptoms
	Passive EAE	Rodents	Transfer of MBP-specific CD4^+^ T cells	Inflammatory cell infiltration, axonal damage and demyelination	Evaluates the mechanisms controlling immune surveillance, effector phase of disease and T-cell-mediated neuroinflammation	This model is not useful for studying relapsing-remitting MS, cannot be used to show remyelination and does not allow evaluating B cell activity in the pathogenesis of the disease
NMOSD	*In vitro*	Astrocytes	AQP4-IgG	Inflammation, changes in astrocytic phenotype and morphology, astrocyte damage, necrosis and AQP4 internalization	Evaluates the pathogenicity of AQP4-IgG, cytotoxicity and cell death. It can also be used for screening drugs	Cannot be used to evaluate demyelination nor to study the relationship between inflammation and demyelination
	*Ex vivo*	Rodents Tissue	AQP4-IgG or AQP4-IgG + complement	Loss of AQP4/GFAP and myelin	This model allows evaluating demyelination, screening drugs and investigate the influence of inflammatory mediators soluble factors involved in the NMOSD	Requires the presence of human complement to be more efficient. Only the isolated antibody is not capable causing demyelinating lesions
	Passive Transfer in EAE Models	Rodents	MBP and CFA (EAE) + AQP4-IgG	Loss of immunoreactivity of AQP4 and GFAP, deposition of IgG and activated complement, granulocyte and macrophages influx, microglial activation and astrocyte injury	It is a consolidated model for the reproduction of typical NMOSD characteristics and useful for investigating mechanisms involved in the early stages of lesion formation. Also important to the study of NMOSD pathogenesis and therapeutics	In this model it is difficult to evaluated demyelination. Axonal damage is not observed, and the lesions in rodents are restricted to the cortical region, different from humans. In addition it requires a large amount of AQP4-IgG (approximately 10 mg) for a single animal injection
	Intracerebral Injection	Rodents	AQP4-IgG or AQP4-IgG + complement	Loss of AQP4, GFAP and myelin, infiltration of mononuclear and polymorphonuclear, glial cell edema, complement deposition, extensive demyelination, early axonal injury and neural cell death	These models requires small amounts of purified AQP4-IgG to be executed, thus are an excellent tool for the study of new drugs and small molecules that can inhibit AQP4-IgG binding to its target	In the mice model, it is necessary a co-administration of human complement to reproduce the disease. Repeated injections of purified AQP4-IgG into the cerebral hemisphere can alter the susceptibility of the CNS to react to additional inflammatory stimuli
	Intrathecal Injection	Rodents	AQP4-IgG	Intraspinal IgG deposition, loss of AQP4 immunoreactivity, astrogliosis, macrophages infiltration, loss of myelin and axons, and loss of GLT-1 and GLAST expression	This model caused lesions independent of complement and reproduces clinical characteristics similar to human as myelopathic signs. It can also be useful to investigate the role of glutamatergic excitotoxicity in the NMOSD	Microglial activation, inflammation, massive infiltration of inflammatory cells and deposition of activated complement components, typical characteristics of human disease are not reproduce in this model
NMDAR Encephalitis	*In vitro*	Neurons	GluN1-IgG	Reduction in NMDA receptor density, receptor internalization and decrease in synaptic currents	This model is useful for evaluating NMDA receptor functionality and expression, as well as for the study of new therapies	This model is not useful for morphological, cytotoxicity, viability and cell death studies, as no other changes are seen
	Passive Transfer	Rodents	GluN1-IgG or CSF of NMDARE patients	Reduction of NMDA receptor density, decrease in synaptic currents, increase in extracellular glutamate levels and in neuronal excitability	The animals present memory impairment, behavioral changes, seizures, and depression, characteristics observed in human NMDAR encephalitis	Cellular and structural changes are difficult to detect in the passive transfer model. Symptoms such as locomotors changes, signs of anxiety, aggressive behavior, spasms, or coma do not occur in this model
	Active Immunization	Rodents	GluN1 peptides or tetrameric GluN1/GluN2B assembled in liposomes	BBB disruption, inflammation and infiltration of peripheral immune cells as pan-leukocyte, activated macrophages, plasma cells, CD4^+^ T cells and B cells	The animals present clinical symptoms and histopathological features similar to humans. Thus, this model allows us to evaluate the course of the disease, the role of specific immune components and the potential for new therapies	Only the GluN1 peptides were not sufficient to mimic the clinic. An immune cascade is necessary for the development and detection of clinical features observed in human NMDAR encephalitis.

*ADEM, acute disseminated encephalomyelitis; AQP4, aquaporin-4; AQP4-IgG, antibodies against AQP4; BBB, blood-brain barrier; CFA, complete Freund's adjuvant; CNS, central nervous system; CSF, cerebrospinal fluid; EAE, experimental autoimmune encephalomyelitis; GLAST, glutamate/aspartate transporter; GLT-1, glutamate transporter-1; GluN1, ionotropic glutamate receptor subunit NR1; GluN1-IgG, antibodies against NR1 subunit; GluN2B, ionotropic glutamate receptor subunit NR2 subtype B; GFAP, glial fibrillary acidic protein; IgG, immunoglobulin G; IFA, incomplete Freund's adjuvant; MS, multiple sclerosis; MAG, myelin-associated glycoprotein; MBP, myelin basic protein; NMOSD, neuromyelitis optica spectrum disorders; NMDA, N-methyl-D-aspartate receptor; NHP, nonhuman primate; PLP, proteolipid protein; rhMOG, recombinant antibodies against MOG; MOG, Myelin oligodendrocyte glycoprotein*.

## MOG-associated Diseases

The myelin oligodendrocyte glycoprotein (MOG) is a protein express on the surface of the myelin sheath. The MOG function is related to cell adhesion, oligodendrocyte microtubule stability and regulation of the complement system (131). In the last few years, the conformational sensitive MOG-IgG antibodies have been widely studied mainly due to it is association to CNS inflammatory injuries such as ADEM, ON, TM, pediatric demyelinating disorders, AQP4-seronegative NMOSD and NMDAR encephalitis with overlapping demyelinating syndromes (132, 133). The development of new techniques such as cell-based assay (CBA) and also the use of new proposed diagnostic classifications of inflammatory disorders, positive MOG-IgG patients may belong to a new clinical entity, distinct from MS and NMOSD (134, 135).

Despite these findings, experimental models of diseases associated with MOG-IgG, excluding the EAE model, have not yet been proposed. In this context, here we suggest that the double transgenic mouse model, initially developed for NMOSD, may be useful to investigate the pathophysiology of MOG associated-diseases.

### Double Transgenic Mouse for MOG-Selective T and B Cell Receptor

B and T cells are important in the pathophysiology of MOG-associated diseases. Both are specific for MOG epitopes and can trigger an inflammatory response of the spinal cord and optic nerves in mice (136, 137), associated with IgG1 antibody (138). TCR and BCR double transgenic models were developed in C57BL/6 mice expressing a MOG-selective T and B cell receptor (TCR-MOG or BCR-MOG, respectively). This model is a valuable tool to investigate the behavior of self-reactive T cells, their interactions with their antigenic targets, and their participation in disease (136). Also, this model demonstrates that the expression of MOG-specific BCR promotes T-cell activation, triggering a pro-inflammatory encephalitogenic process even without antibody production (139, 140). Thus, there is a cooperation between T and B cells, T cells induce active production of MOG-specific IgG1 antibody and MOG-specific B cells increase MOG-specific T cell proliferation and activation (136).

The double transgenic model was initially developed to reproduce NMOSD. The CNS lesions were mainly observed in the optic nerve and spinal cord, suggesting that this model would be the best to resemble human pathogenesis. However, at the cellular and molecular level, the complement, perivascular IgM, IgG and anti-AQP4 antibodies related to human NMOSD disease were not found in this double transgenic mouse. Furthermore, as described by Krishnamoorthy et al. (137), inflammatory cells such as neutrophils and eosinophils were absent (137). Thus, the double transgenic mouse cannot reproduce NMOSD pathology; however, this model can be used to investigate MOG-associated diseases, as it provides an important and clear relation to understanding antigen-specific B cell and T cell crosstalk.

## From Preclinical to Clinical Research

Translational research is the capacity of transforming observations from preclinical and clinical studies into interventions that improve the health of individuals. Among all the experimental models described in this review article, the EAE animal model is undoubtedly the one that has contributed most to understanding MS pathogenesis and novel therapies for this disease. Indeed, drugs such as glatiramer acetate, dimethyl fumarate, teriflunomide, daclizumab, alemtuzumab and mitoxantrone are classic examples of EAE studies translated into clinical practice (54, 141). In contrast, there are several failures in this model. An important example consists of the role of TNF in EAE and MS. Preclinical studies have shown that inhibition of TNF signaling improves the course of the disease. However, treatment with infliximab—a TNFR blocker—worsened MS symptoms (142, 143). The same applies to the blockage of the transcriptional factor BAFF and pro-inflammatory cytokine IL-23, whose effects are pivotal in suppressing EAE, whereas no activity was observed in patients with relapsing-remitting MS (144).

Failure to translate preclinical outcomes into clinical therapies has been partially attributed to the experimental design, including internal and external validity. Thus, Vesterinen et al. performed a systematic review of EAE experimental design using articles from 1961 to 2008. The authors concluded that of the 1,117 studies analyzed, only 9% were randomized, 10% were blinded and 1% included power calculation (145). Therefore, in order to have higher translational efficacy in the *in vivo* research of neuroimmunology, the implementation of good experimental practice, such as the experimental design, is fundamental to reduce the risk of biased results. Nowadays, the National Centre for the Replacement, Refinement & Reduction of Animals in Research (NC3Rs) provides the Experimental Design Assistant (EDA), a free online guide that can be used by researchers to calculate the minimum number of animals required for their studies, to reduce experimental biases and to choose appropriate statistical analysis.

Of note, there is no single animal model that can mirror the whole spectrum of any human disease. The first step is to think which question we want to answer, for example: Does this compound have remyelination activity? What is the immunopathological mechanism of the disease? Is neurodegeneration involved? After that, we must choose the best way to answer the question, balancing the limitations of the *in vitro* or *in vivo* models.

A decade ago, the prognosis of a patient with NMOSD was very poor. The correct questions and the use of appropriate methodology allowed distinguishing MS from NMOSD, in addition to elucidating the cellular and molecular mechanisms, such as seropositive AQP4-specific antibodies, complement participation, encephalitogenic T cell, neutrophil and cytokine crosstalk in the lesions. This has led to clinical studies with monoclonal antibodies that interfere in specific parts of the immune system associated with NMOSD, as well as the development of a monoclonal antibody that competes with AQP4-IgG called aquaporumab (146).

In conclusion, ongoing research focusing on developing experimental models of neuroimmunological diseases is expected to provide a better comprehension of critical topics, i.e., immunopathogenic signaling and novel therapeutic approaches. Moreover, to maximize the reproducibility of preclinical experiments, the Animal Research: Reporting *In Vivo* Experiments (ARRIVE) guideline should be used to describe each step of the study. Finally, *in vitro* and *in vivo* studies are vital to understand the biology of neuroimmunological disorders and to develop innovative drugs in the future.

## Challenging Frontiers in Neuroimmunological Models

In recent years, the preclinical models of neuroimmunological diseases have advanced significantly. A broad spectrum of animal models is currently available to cover some gaps. Conversely, there are key aspects of human diseases that are not elucidated because of technical limitations. Examples in experimental MS include the following: (i) the role of B cells and CD8^+^ T cells; (ii) mechanisms of demyelination; and (iii) progressive stage of MS. In addition, environmental factors such as vitamin D and bacterial infection should be considered. Therefore, new *in vivo* models must be developed to address these questions.

Concerning novel animal models, zebrafish is an emerging example. It can be used for many neurodegenerative diseases, including MS and other demyelinating diseases (147). Zebrafish reproduces rapidly, generates many embryos and thus becomes an excellent tool to validate potential therapies from primary screens, something that is impossible to do today with other animal models. Furthermore, the myelination/demyelination process can be assessed in real time through genetic mutations, including cell-cell crosstalk mainly by oligodendrocyte precursor cell differentiation (148). Recently, an EAE zebrafish model has been developed, resulting in paralysis, reduced body weight, microglial influx and reduced survival, i.e., the same parameters observed in EAE mouse model. The main advantage is that the symptoms are detected 3 days after immunization (141). Nonetheless, zebrafish models are at an early stage of characterization, and further studies are needed to evaluate the involvement of their immune system in myelin damage.

There is an urgent need to develop novel approaches to successfully treat various neuroimmunological diseases. In this context, clustered regularly interspaced short palindromic repeats/CRISPR-associated protein 9 (CRISPR/Cas9) has proven to be a powerful tool for inducing gene correction, disease modeling, transcriptional regulation, epigenome engineering, chromatin visualization as well as for developing neurotherapies through the genome, RNA and epigenome editing. The innate and adaptive immune response added to microglia and astrocytes is a key cellular mediator of neuroinflammation. Hence, we believe that precision-targeted genome editing of key signaling molecular mechanisms underlying neuroinflammation offers a novel therapeutic approach to effectively treat neuroimmune disorders. Currently, this technique has been applied to neurodegenerative diseases, especially Alzheimer's disease, Parkinson's disease, amyotrophic lateral sclerosis and Huntington's disease. Furthermore, the environmental factors involved in NMOSD should be investigated, mainly when pathogens such as *Helicobacter pylori* and *Clostridium spp*. are present. The use of tools such as MRI, visual-evoked potential (VEP) and optical coherence tomography (OCT) might contribute significantly to monitor the disease in a preclinical setting.

Lastly, the humanized mouse technology, i.e., immunodeficient mice engrafted with functional human cells and tissues, could be an interesting strategy to evaluate the efficacy and safety of drug candidates and signaling cells in neuroimmunology. This approach is being widely used as *in vivo* models in different biological fields such as infectious diseases, immunology, cancer, regenerative medicine, hematology and autoimmunity (149). Hence, understanding the humoral and adaptive immune system, mainly B cells, could accelerate a breakthrough in the field of neuroimmunology. Additionally, the exchange of knowledge between neurologists, pathologists and basic scientists may open new avenues in the neuroimmune experimental models, thereby changing the *status quo*.

## Author Contributions

All authors contributed to the manuscript preparation and wrote, read and approved the submitted version.

## Conflict of Interest

AS, LG, and RM have received scholarships from CAPES/Brazil. RS is a pharmacology post-doctoral researcher receiving grants from the National Institute of Science and Technology in Tuberculosis (INCT-TB/CNPq). DS has received a scholarship from the Ministry of Education, Culture, Sports, Science and Technology (MEXT) of Japan; a Grants-in-Aid for Scientific Research from the Japan Society for the Promotion of Science (KAKENHI 15K19472); research support from CNPq/Brazil (425331/2016-4), FAPERGS/MS/CNPq/SESRS (17/2551-0001391-3) PPSUS/Brazil, TEVA (research grant for EMOCEMP Investigator Initiated Study) and Euroimmun AG (Neuroimmunological Complications associated with Arboviruses); has received speaker honoraria from Biogen, Novartis, Genzyme, TEVA, Merck-Serono, Roche and Bayer and has participated in advisory boards for Shire, Roche, TEVA, Merck-Serono and Quest/Athena Diagnostics. The remaining author declares that this research was conducted in the absence of any commercial or financial relationships that could be construed as a potential conflict of interest.
